# A Fusion Protein between Streptavidin and the Endogenous TLR4 Ligand EDA Targets Biotinylated Antigens to Dendritic Cells and Induces T Cell Responses *In Vivo*

**DOI:** 10.1155/2013/864720

**Published:** 2013-09-05

**Authors:** Laura Arribillaga, Maika Durantez, Teresa Lozano, Francesc Rudilla, Federico Rehberger, Noelia Casares, Lorea Villanueva, Marta Martinez, Marta Gorraiz, Francisco Borrás-Cuesta, Pablo Sarobe, Jesús Prieto, Juan José Lasarte

**Affiliations:** ^1^Gene Therapy and Hepatology Area, Center for Applied Medical Research (CIMA), University of Navarra, 31008 Pamplona, Spain; ^2^CIBERehd, Pamplona, Spain

## Abstract

The development of tools for efficient targeting of antigens to antigen presenting cells is of great importance for vaccine development. We have previously shown that fusion proteins containing antigens fused to the extra domain A from fibronectin (EDA), an endogenous TLR4 ligand, which targets antigens to TLR4-expressing dendritic cells (DC), are highly immunogenic. To facilitate the procedure of joining EDA to any antigen of choice, we have prepared the fusion protein EDAvidin by linking EDA to the N terminus of streptavidin, allowing its conjugation with biotinylated antigens. We found that EDAvidin, as streptavidin, forms tetramers and binds biotin or biotinylated proteins with a *K*_*d*_ ~ 2.6 × 10^−14^ mol/L. EDAvidin favours the uptake of biotinylated green fluorescent protein by DC. Moreover, EDAvidin retains the proinflammatory properties of EDA, inducing NF-**κ** **β** by TLR4-expressing cells, as well as the production of TNF-**α** by the human monocyte cell line THP1 and IL-12 by DC. More importantly, immunization of mice with EDAvidin conjugated with the biotinylated nonstructural NS3 protein from hepatitis C virus induces a strong anti-NS3 T cell immune response. These results open a new way to use the EDA-based delivery tool to target any antigen of choice to DC for vaccination against infectious diseases and cancer.

## 1. Introduction

The development of prophylactic and therapeutic vaccines requires strategies capable of stimulating CD8^+^ cytotoxic T cells which recognize antigens expressed by infected cells or tumors. The unique capacity of DC to present antigens to T cells and elicit immune responses has prompted their use in vaccination strategies. The “*in vivo* loading” or “targeting” of antigens to DC through their surface receptors [1–9] constitutes an alternative to the *ex vivo* manipulation of dendritic cells (DC) for their transfer into the patient (reviewed in [10]). It has been described that the efficacy of antigen capture by DC dramatically affects the immunogenicity of the antigen. But, the outcome of the immune response induced by targeting antigens to DC depends on the receptor used [3, 11–15]. Engagement of TLR on DC loaded with the antigen induces DC activation, expression of cytokines, and migration to draining lymph nodes for an efficient presentation of the processed antigen to T cells [16]. Recently, we have demonstrated that fusion of an antigen to the extra domain A from fibronectin (EDA) favours antigen targeting to TLR4-expressing DC, leading to their maturation and enhancing cross-presentation and immunogenicity of the antigen [4] as well as the induction of antiviral/tumor immunity [17–19]. However, this vaccination strategy requires the preparation of the corresponding fusion protein between EDA and the selected antigen each time. In order to facilitate the procedure of joining EDA to different viral or tumoral antigens, we have taken the advantage of the exceptionally high affinity of streptavidin for biotin, one of the strongest known noncovalent biological interactions (*K*_*d*_ > 10^−15^ M) [20]. We hypothesised that a fusion protein of EDA linked to streptavidin could be easily conjugated to biotinylated antigens for their use as immunogens for vaccination purposes. In this work, we found that EDAvidin was able to tetramerize and bind to biotinylated proteins while retaining the proinflammatory and DC targeting properties of EDA. In addition, we found that EDAvidin conjugated with the NS3 protein from hepatitis C virus (HCV) was able to induce strong and specific T cell immune responses against the main T cell epitopes from NS3 protein.

## 2. Material and Methods

### 2.1. Construction and Purification of the EDAvidin

The plasmid pET21a-Streptavidin-Alive [21] (Addgene, Cambridge, MA) expressing wild-type subunit of streptavidin with a 6xHis tag was used for the construction of the expression plasmid pET21a-EDA-Streptavidin using conventional cloning techniques. This plasmid expressing EDA in the C-terminal end of streptavidin was verified by DNA sequencing and introduced into BL21(DE3). Recombinant protein EDAvidin was purified from inclusion bodies by affinity chromatography (Histrap, GE Healthcare, Uppsala, Sweden), refolded in a sepharose G25 column using a urea gradient size-exclusion chromatography, dialysed, and removed from endotoxins by using EndoTrap columns (Profos AG, Regensburg, Germany) until endotoxin levels were below 0.2 EU/*μ*g protein (tested by quantitative chromogenic limulus amebocyte lysate assay (Cambrex, Walkersville, MD, USA)). Purified recombinant protein was analyzed by SDS-PAGE and stained with Coomassie blue (Bio-Safe Coomassie reagent, Bio-Rad, Hercules, CA, USA). 

### 2.2. Binding Assay of EDAvidin to Biotinylated Proteins by SDS-PAGE

The molecular weight marker containing biotinylated proteins (MW 6,500–180,000, Sigma), or the High-Range Rainbow Molecular Weight Marker (12000–225000, GE Healthcare) as negative control, were loaded into a 10% SDS-PAGE, transferred to nitrocellulose membranes, and incubated with 1.33 nmol of EDAvidin or EDA. After washing, membranes were incubated with a rabbit polyclonal anti-EDA antibody (1/500) produced in our laboratory. Membranes were then incubated with anti-rabbit IgG horseradish-peroxidase (Cell Signaling) antibody (1/2500) and developed using ECL chemiluminescence system (Amersham). As a positive control, a membrane was incubated with horseradish peroxidase conjugated streptavidin (1/500) (GE Healthcare). 

In some experiments, EDAvidin was incubated with biotinylated proteins (i.e., OVA biot) and loaded into a 10% SDS-PAGE to analyze the molecular size of the complexes formed in comparison with the monomer OVA or the EDAvidin tetramer alone. Gels were stained with Coomassie blue (Bio-Safe Coomassie, Hercules, CA, USA).

### 2.3. ELISA-Based Binding Assays of EDAvidin to Biotinylated Proteins

OVA protein (grade III), BSA (Sigma), or the nonstructural NS3 protein from hepatitis C virus [19] were biotinylated using sulfo-NHS-SS-Biotin (Thermo Scientific) following manufacturer's instruction. Microtiter plates (Nunc MaxiSorp, Roskilde, Denmark) were coated with 0.1 *μ*g/well of biotinylated proteins in carbonate buffer 0.1 M (pH 9,5). Then, plates were incubated with PBS containing 10% FCS (blocking buffer) during 1 h at room temperature. After removing the blocking buffer, a 1/500 dilution of EDAvidin or EDA protein was added and incubated at 37°C for 90 minutes, washed, and incubated at 37°C for 1 h with a 1/500 dilution of a rabbit polyclonal anti-EDA antibody followed by a 1/2500 dilution of anti-rabbit whole IgG horseradish peroxidase conjugated antibody (Sigma). Plates were developed by adding 100 *μ*L of TMB (BD Biosciences) and read at 450 nm using the Multiskan Ascent (Thermo Electron Corporation). 

### 2.4. Biomolecular Interaction Analysis

Binding capacity of EDAvidin to biotinylated proteins was also analyzed by surface plasmon resonance (SPR) using ProteOn XPR36 (Bio-Rad, Hercules, CA, USA) optical biosensor. OVA and OVA-biotinylated proteins were covalently immobilized onto the surface of a GLC sensor chips (Bio-Rad) using the coupling reagents sulfo-NHS and EDC (Bio-Rad). After protein immobilization, chip surface was treated with ethanolamine to deactivate the excess of reactive esters. To determine the equilibrium dissociation constant (*K*_*d*_) of EDAvidin to biotinylated OVA protein, different concentrations of EDAvidin with 2-fold dilution of the maximum concentration 100 nM were injected in running buffer (PBS, 0.005% (v/v) Tween 20, pH 7.4) over the coated sensor ships at a flow rate of 30 *μ*L/min. Protein binding was evaluated during an association phase (0–300 sec), which was followed by a dissociation phase (injection of buffer only, 300–3700 sec). The association phase, where EDAvidin protein is flowed across the OVA biot coated sensor ship and binding is measured, allows the determination of the rate of formation of the complex over the time which is reflected by an increase in the SPR response units (RUs). The kinetic of the increase in RU determines the association constant (*K*_*a*_). In the dissociation phase, the EDAvidin protein is removed from the flow, and the rate of complex dissociation follows exponential decay kinetics. This kinetic determines the dissociation constant (*K*_*d*_). Data were double referenced by subtraction of control flow cell (coated with nonbiotinylated OVA) and data from Interspots, as recommended by the manufacturers. EDAvidin affinity to OVA-biot and rate constants of the interaction was determined by global analysis using Langmuir binding model provided by the ProteOn X36 software (Bio-Rad). After this process, the chip surface was regenerated by the injection of free biotin (2 *μ*M), to remove the EDAvidin coated protein to the chip. After this regeneration process, different concentrations of streptavidin were injected in running buffer at a flow of 30 *μ*L/min to determine the *K*_*d*_ for streptavidin-biotin interaction. 

### 2.5. Mice

Female C57BL/6 mice, 6–8 weeks old, from Harlan (Barcelona, Spain) and HHD mice, transgenic for human HLA-A2.1 and beta-2 microglobulin molecules [22], kindly provided by Dr. F. Lemonnier (Institute Pasteur, Paris, France), were housed in appropriated animal care facilities during the experimental period and handled following the local and international guidelines required for experimentation with animals.

### 2.6. Targeting of Antigen to DC

To study the targeting capacity of EDAvidin to DC, recombinant green fluorescent protein (GFP) was biotinylated as described above and incubated for 15 min with EDAvidin or with EDA. Resulting mixtures were incubated with C57BL/6-derived bone-marrow-derived DC (BMDC) [4] for 15 min at 4°C, washed, and analyzed by flow cytometry. To study the capacity of EDAvidin to stimulate the production of IL-12 by DC, BMDC were cultured at 37°C and 5% CO_2_ with EDA or EDAvidin (500 nM), LPS (0.1 *μ*g/mL), or culture medium. One day later, supernatants were harvested to measure, and IL-12 (p70) by ELISA (BD-Pharmingen), according to manufacturer's instructions. 

### 2.7. *In Vitro* Analysis on Monocyte Activation and Measurement of Activation of TLR4 Signaling Pathway

THP-1 cells (ATCC, Manassas, VA, USA) were grown as described [19], plated at 2 × 10^5^ cells/well, and cultured in the presence of different concentrations of the indicated antigens. After 15 hours of incubation, culture supernatants were harvested and human TNF-*α* released to the medium was quantified using a commercial ELISA assay (BD-Pharmingen), according to manufacturer's instructions. To measure activation of TLR4 signaling pathway, HEK293/hTLR4-MD2-CD14 or HEK293/LacZ expressing cells were transfected with plasmid pNiFty-SEAP (Invivogen) carrying the human secreted embryonic alkaline phosphatase gene (SEAP) controlled by an NF-*κβ*-inducible ELAM-1 promoter. Twenty-four hours after transfection, cells were incubated in the presence or absence of different concentrations of the indicated molecule. After 24 hours, the expression of the reporter gene was measured in culture supernatants by a colorimetric assay (SEAP reporter assay kit, Invivogen). Results represent the fold NF-*κβ* induction factor (OD obtained with supernatants from HEK293/TLR4-MD2-CD14 divided by OD obtained with supernatants from HEK293/LacZ).

### 2.8. *In Vivo* Induction of Anti-NS3 Immune Responses after Immunization with EDAvidin Plus Biotinylated HCV-NS3 Protein

HHD mice [22] were immunized i.v. with 200 *μ*L of a saline solution containing (i) 2 nmol of EDAvidin plus biotinylated NS3, (ii) 2 nmol of EDA-NS3, (iii) 2 nmol of biotinylated NS3, (iv) 2 nmol of EDA plus 2 nmol of biotinylated NS3, and (v) 2 nmol of streptavidin plus 2 nmol of biotinylated NS3. Seven days after immunization, cytotoxic T cell activity (CTL activity) was measured by an *in vivo* killing assay. Briefly, naive splenocytes from HHD mice were pulsed with the HLA-A2-restricted peptide p1073 (CVNGVCWTV) from NS3 (10 *μ*g/mL; 30 minutes, 37°C), washed extensively, and labeled with a high concentration (1.25 *μ*M) of CFSE (Invitrogen). The nonpulsed control population was labeled with a low concentration (0.125 *μ*M) of CFSE. Both CFSE^high^- and CFSE^low^-labeled cells were mixed at a 1 : 1 ratio (5 × 10^6^ cells of each population) and then injected intravenously into immunized mice. The number of CFSE+ cells remaining in the spleen after 20 hours was determined by flow cytometry, and the specific lysis was calculated as previously described [23]. T cells producing IFN-*γ* were enumerated by ELISPOT using a kit from BD-Biosciences (San Diego, CA, USA) according to manufacturer instructions, by culturing 8 × 10^5^ splenocytes from immunized mice in the absence/presence of peptide p1073 (10 *μ*g/mL), NS3 protein (0.1 *μ*g/mL), or culture medium (negative control). The number of spots was counted using an automated ELISPOT reader (CTL, Aalen, Germany).

### 2.9. Statistical Analysis

Normality was assessed with Shapiro-Wilk *W* test. Statistical analyses were performed using parametric (Student's *t*-test and one-way ANOVA) and nonparametric (Kruskal-Wallis and Mann-Whitney *U*) tests. For all tests, a *P* value < 0.05 was considered statistically significant. Descriptive data for continuous variables are reported as means ± SEM. Prism software (GraphPad Software, Inc.) was used for statistical analysis.

## 3. Results

### 3.1. Recombinant EDAvidin Tetramerizes and Binds to Biotinylated Proteins

**Figure 1:**
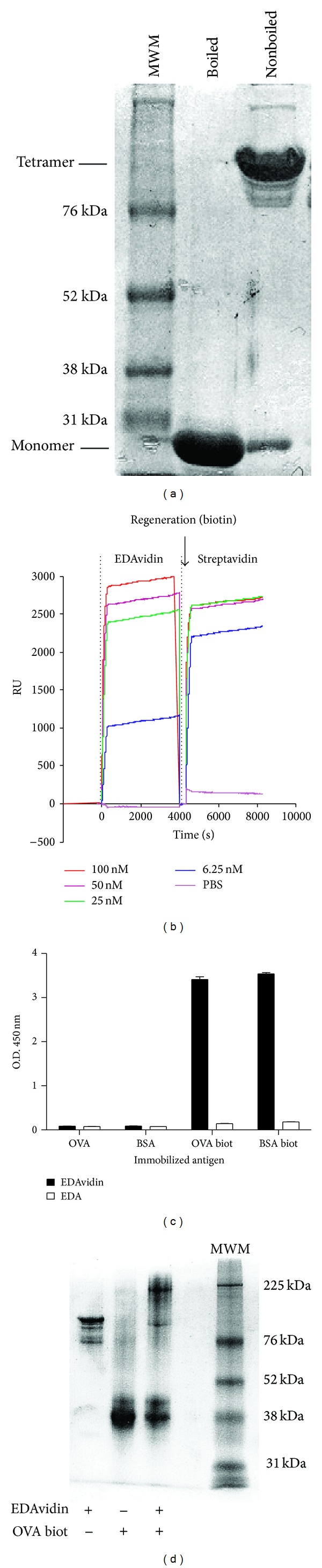
Recombinant EDAvidin tetramerizes and binds to biotinylated proteins. (a) SDS-PAGE of purified recombinant proteins stained with Coomassie blue (1: (MWM: molecular weight marker); 2: denatured EDAvidin; 3: nondenatured EDAvidin). (b) Surface plasmon resonance analysis of the capacity of EDAvidin and streptavidin to bind biotinylated proteins. Biotinylated ovalbumin was coated into the chip, and EDAvidin or streptavidin were injected at different concentrations. The surface of the chip was regenerated by the injection of an excess of 2 *μ*M biotin before the injection of streptavidin (RU: surface plasmon resonance response units). (c) ELISA-based binding assays of EDAvidin to biotinylated proteins. Biotinylated or nonbiotinylated ovalbumin (OVA) and bovine serum albumin (BSA) were coated into the wells of ELISA plates. EDAvidin or EDA alone was added to the wells and after extensive washes, the plates were developed using rabbit polyclonal anti-EDA antibodies. (d) Binding assay of EDAvidin to biotinylated proteins by SDS-PAGEas 1: EDAvidin in its tetrameric form; 2: biotinylated OVA; 3: EDAvidin plus biotinylated OVA; 5: (MWM).

Several reports have shown that the strong affinity of avidin or streptavidin for biotin is dependent upon the tetrameric architecture of the protein [24]. We produced the recombinant protein EDAvidin by linking EDA to the C-terminal end of streptavidin. SDS-PAGE analysis of the purified protein showed a band corresponding to the putative molecular weight of a tetramer form of EDAvidin (98 kDa) (Figure 1(a)). When the sample was boiled before the SDS-PAGE analysis, a band corresponding to the monomer was observed (24.5 kDa), suggesting that the fusion protein tetramerizes spontaneously in solution. 

We studied by surface plasmon resonance the capacity of EDAvidin to bind to the surface of a chip coated with biotinylated OVA. Thus, by using different EDAvidin concentrations (100–6,5 nM), we found that EDAvidin bound with high affinity to biotinylated OVA protein (Figure 1(b)). When comparing this binding with that of streptavidin, we observed that EDAvidin had a slightly lower affinity (*K*_*d*_ ~ 2.3 × 10^−14^ mol/L), although it is still in the range of the very high affinity constant showed by streptavidin (*K*_*d*_ > 10^−15^ mol/L). 

Binding of EDAvidin to biotinylated proteins OVA (OVA biot) or BSA (BSA biot) was also studied by ELISA. By incubating OVA biot or BSA Biot-coated plates with EDAvidin or with EDA (as control) and by quantification with anti-EDA antibodies, it was found that EDAvidin, but not free EDA, bound to biotinylated proteins (Figure 1(c)). The binding capacity of EDAvidin to biotinylated proteins was also studied by SDS-PAGE. Thus, when EDAvidin was mixed with OVA biot, the tetrameric EDAvidin was converted into a larger molecular complex, corresponding to the putative EDAvidin-OVA biot association (Figure 1(d)). Indeed, a band shift with a retardation in the gel was observed for the tetrameric EDAvidin (lane 1) or for the free OVA biot (lane 2) when both proteins were combined (lane 3). Although we have not done the stoichiometric analysis, the larger molecular complex found in lane 3 should correspond to the putative molecular weight of 3 or 4 molecules of bOVA combined with the tetrameric EDAvidin. Binding of EDAvidin to biotinylated proteins was also studied by Western-blot using anti-EDA antibodies to detect a molecular weight marker mixture consisting in biotinylated proteins. It was found that only EDAvidin bounds to the biotinylated proteins (See Supplementary Figure  1 in Supplementary Material available online at http://dx.doi.org/10.1155/2013/864720).

### 3.2. EDAvidin Retains the Proinflammatory Activity of EDA and Targets Biotinylated Antigens to DC

**Figure 2:**
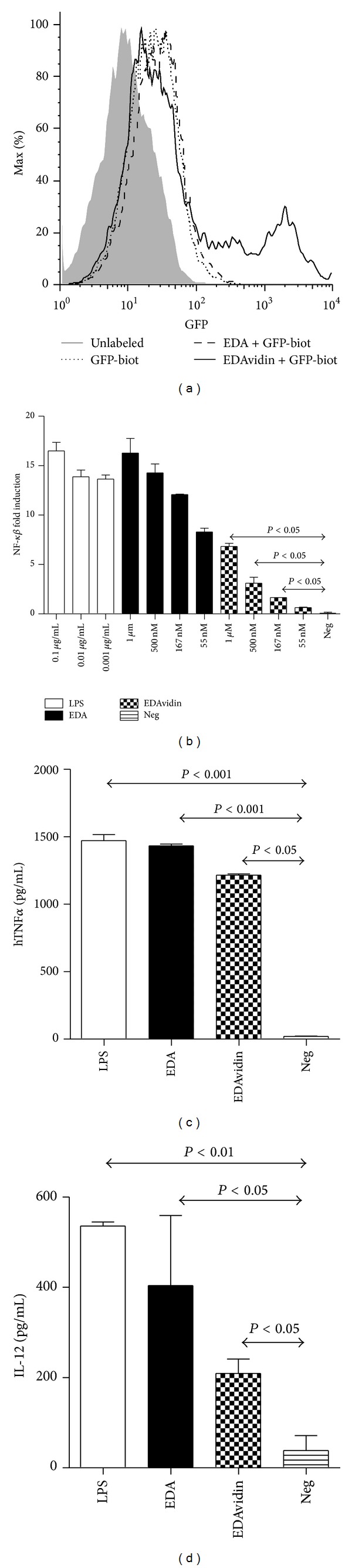
EDAvidin targets biotinylated antigens to DC and retains the proinflammatory activity of EDA. (a) Flow cytometric analysis of BMDC incubated with the indicated proteins. (b) Colorimetric assay to measure NF-*κβ* induction in HEK TLR4 or HEK LacZ expressing cells in response to different concentrations of EDA, EDAvidin, LPS, or culture medium (Neg). Results represent the NF-*κβ* fold induction. (c) The human monocytic cell line THP1 was incubated in the absence or presence of the indicated concentrations of EDAvidin, EDA, LPS, or culture medium (Neg). After 15 hours of culture, supernatants were harvested and the released TNF-*α* was measured by ELISA. (d) Production of IL-12 by BMDC after incubation with 500 nM EDA, 500 nM EDAvidin, 0.1 *μ*g/mL LPS or culture medium (Neg). Twenty-four hours later, culture supernatant was harvested and IL-12 released to the medium was measured by ELISA. All data are representative of two independent experiments.

In previous works, we found that recombinant EDA bound to TLR4 and activated its downstream signaling pathway [4, 19]. To study the capacity of EDAvidin to target an antigen to DC, biotinylated green fluorescent protein (GFP-biot) was mixed with EDAvidin and added to BMDC. After 15 min of incubation, cells were washed, and GFP uptake by DC was analyzed by flow cytometry. It was found that a significant proportion of BMDC incubated with EDAvidin + GFP-biot was highly labeled with the fluorescent protein (Figure 2(a)). This result was not found when BMDC were incubated with biotinylated GFP alone or in combination with EDA, suggesting that EDAvidin was targeting the biotinylated protein to DC.

EDA activates TLR4 signaling pathway and induces DC maturation and the production of proinflammatory cytokines by activated cells [4]. We thus first analyzed EDAvidin-induced TLR4 signaling, measured as translocation of NF-*κβ*. By using HEK TLR4 or HEKLacZ cells transfected with a plasmid carrying the human secreted embryonic alkaline phosphatase gene, under the control of the NF-*κβ*-inducible ELAM-1 promoter, we observed that EDAvidin, although less efficient than EDA, activated the TLR4 signaling pathway (Figure 2(b)).

We next studied the proinflammatory activity of EDAvidin by measuring its capacity to induce the production of cytokines in two different systems: TNF-*α* by the TLR4^+^ human monocytic cell line THP1 and IL-12 by murine BMDC. In both cases, we observed that EDAvidin, although less efficient than free EDA, induced the production of the corresponding cytokine (Figures 2(c) and 2(d)). 

### 3.3. EDAvidin Plus Biotinylated NS3 Induces Strong Anti-NS3 Cellular Immune Responses *In Vivo*

**Figure 3:**
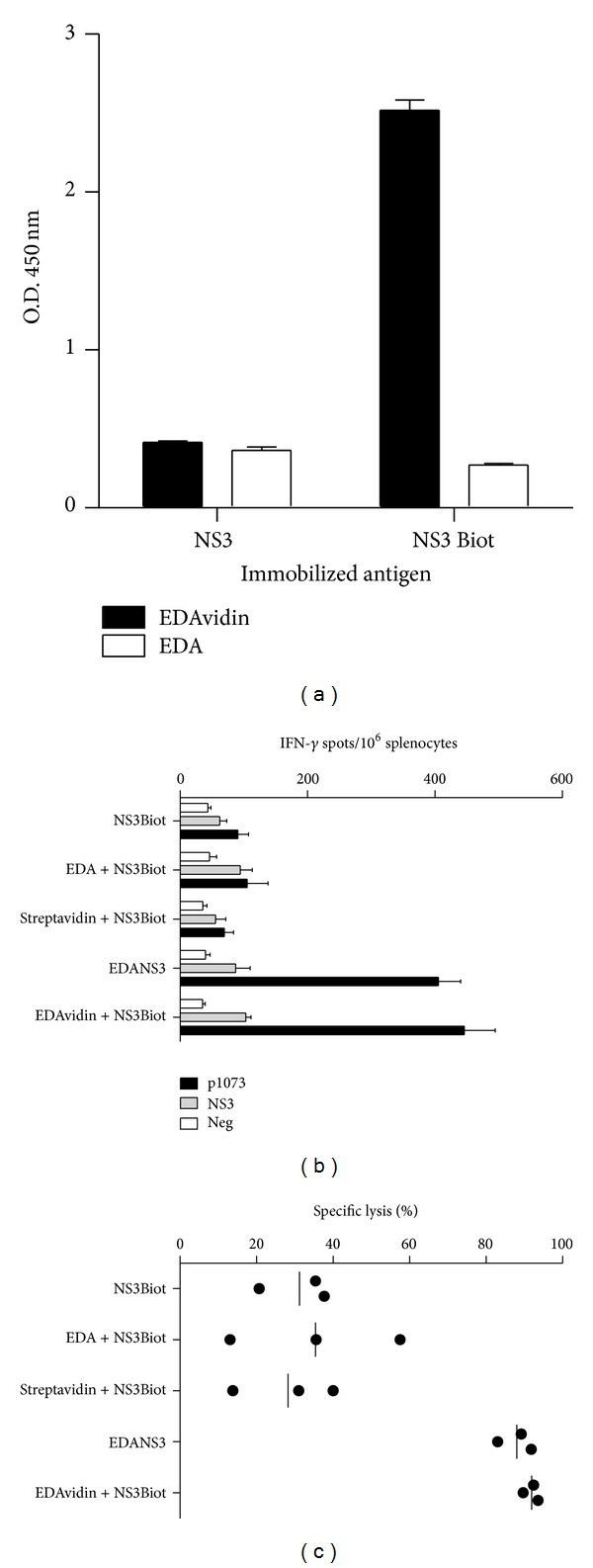
EDAvidin binds to biotinylated NS3 and induces strong anti-NS3 cellular immune responses *in vivo*. (a) ELISA-based binding assays of EDAvidin to biotinylated NS3. Biotinylated or nonbiotinylated NS3 were coated into the wells of ELISA plates. EDAvidin or EDA alone was added to the wells and after extensive washes, the plates were developed using rabbit polyclonal anti-EDA antibodies. ((b), (c)) *In vivo* induction of anti-NS3 T cell responses. HHD transgenic mice were immunized i.v. with NS3Biot, EDA plus NS3Biot, streptavidin plus NS3Biot, EDA-NS3, or with EDAvidin plus NS3Biot in saline. Seven days after immunization, anti-NS3 immune response was analyzed by measuring the number of IFN-*γ* producing cells by ELISPOT in response to the T cell epitope p1073, NS3 protein, or culture medium (Neg) (b) or by *in vivo* killing (c) as described in methods.

We had shown that the fusion protein EDA-NS3, containing NS3 protein from hepatitis C virus, induced strong cellular immune responses specific for NS3 when used as immunogen [19]. Therefore, we sought to analyze its immunogenic properties *in vivo* when administered with a biotinylated antigen. As with previous biotinylated proteins, we first demonstrated that biotinylated NS3 (NS3Biot) coated in an ELISA plate is associated with EDAvidin, but not with free EDA (Figure 3(a)). We then tested *in vivo* the immunogenicity of a mixture of EDAvidin plus NS3Biot by immunizing HHD transgenic mice. Its immunogenicity was compared with equivalent molar amounts (2 nmol) of EDA-NS3, EDA plus NS3Biot, NS3Biot or streptavidin plus NS3Biot. One week after immunization, mice were sacrificed and spleen cells were cultured in the presence of the NS3 HLA-A2-restricted CD8 epitope p1073 or with recombinant NS3 protein to measure the number of IFN-*γ* producing cells by ELISPOT. This experiment showed that EDAvidin plus NS3biot was as good as EDA-NS3 protein to induce anti-NS3 specific T cell immune responses, whereas NS3biot alone or mixed with streptavidin or with free EDA barely induced any response (Figure 3(b)). Similar results were obtained when analyzing T cell responses in *in vivo* killing assays, which measured the capacity of these immunogens to induce cytotoxic T cells able to kill target cells pulsed with peptide p1073 (Figure 3(c)). 

## 4. Discussion

Over the last decades, several vaccine strategies enabling delivery of Ags for presentation by APC have been assayed with varying degrees of success [3, 11–15, 25–28]. Since maturation of the DC is essential to trigger adaptive immune responses [29], procedures that simultaneously target the antigen to DC and induce their maturation could lead to the development of a new generation of vaccines that might work in synergy with mild and safe adjuvants. We have previously reported that fusion of an antigen with EDA leads to antigen targeting to TLR4-expressing DC, enhancing cross-presentation and immunogenicity [4]. Here, we describe a novel antigen delivery approach in which a biotinylated antigen is bound noncovalently to EDAvidin protein, a construct which retains the TLR4 targeting ability and inflammatory properties of EDA. This would allow the combination of the tetrameric EDAvidin with a broad range of commercially available antigens or adjuvants which can be easily biotinylated, facilitating the preparation of DC-targeted antigens to be used as immunogens for the induction of T cell responses.

By using different approaches, we have shown that EDAvidin forms tetrameric complexes and binds to biotinylated antigens with a very high affinity (*K*_*d*_ ~ 2.3 × 10^−14^ mol/L). Importantly, EDAvidin greatly increased biotinylated GFP uptake by DC and retained EDA proinflammatory capacity. It induced NF-*κβ* activation, an important mediator for DC maturation [30, 31] and stimulated the production of TNF-*α* by THP1 cells as well as the production of IL-12 by murine BMDC. The final aim when designing EDAvidin was to facilitate conjugation of EDA to biotinylated antigens to be used as vaccines. Thus, we compared the immunogenicity of EDAvidin plus NS3biot with that of a fusion protein between EDA and HCV NS3, an immunogen known to induce a specific T cell response when administered in the absence of additional adjuvants [19]. We first found that EDAvidin interacted physically with NS3biot. But more importantly, we found that immunization with a mixture of EDAvidin and NS3biot induced a T cell immune response against NS3 similar to that obtained when using EDA-NS3 fusion protein. It is interesting to note that either when NS3 is not linked to the antigen delivery system (e.g., by using EDA instead of EDAvidin), or when tetrameric complexes do not retain the proinflammatory properties of EDA (e.g., when using streptavidin instead of EDAvidin), the immunogens show a much lower efficacy, demonstrating that both EDAvidin properties, antigen targeting, and DC activation are essential for efficient priming of T cells responses. 

Multivalency of streptavidin and EDAvidin might be considered as an advantage for some applications. The tetrameric structure of EDAvidin might allow the combination of different biotinylated antigens or adjuvants within a single targeting vector. This would favour both sides required for antigen presentation, simultaneous targeting, and therefore induction of immune responses against several antigens, as well as the inclusion of new biotinylated adjuvants which would collaborate with EDA in APC activation [17–19], a situation that has been demonstrated to improve phagosome maturation and antigen presentation by APC [32]. 

Proteins can be biotinylated chemically or enzymatically using already established protocols. Using our strategy, we could also consider the possibility to biotinylate more complex antigenic structures such as whole cells for their engineering to codisplay immunomodulatory molecules, as it has been described previously with a different approach [33]. EDAvidin could also be used to decorate a biotinylated tumor cell to render it more immunogenic allowing its capture by TLR4 expressing DC. Future experiments need to be conducted to explore this possibility. 

In summary, we have found that a chimeric protein containing EDA fused to the N terminus of streptavidin retains functional properties of EDA and facilitates its conjugation to any antigen of choice and results in a new tool which opens a new way to use this antigen delivery system in vaccination against infectious diseases and cancer. 

## Supplementary Material

Online Supplemental Material includes a western blot experiment to demonstrate the binding capacity of EDAvidin to biotinylated proteins (Supplementary Figure 1).
Legend for Supplementary Figure 1: EDAvidin binds to biotinylated proteins. A molecular weight marker containing biotinylated proteins (M.W. 6,500-180,000, Sigma) (Biot), or the High-Range Rainbow Molecular Weight Marker (12000-225000, GE Healthcare) (RB) as negative control, were loaded into a 10% SDS-PAGE followed by electrophoretic transfer to nitrocellulose membranes. The detection of biotinylated proteins was carried out by incubating the membranes with 1,33 nmol of EDAvidin or EDA protein. After washing, the membranes were incubated with a rabbit polyclonal anti-EDA antibody. Membranes were them incubated with anti rabbit IgG horseradish-peroxidase (Cell Signalling), and developed by using ECL chemoluminescence system (Amersham). As a positive control, one of the membranes was incubated with a dilution 1/500 of horseradish peroxidase conjugated streptavidin.

## Abbreviations

EDA: Extra domain A from fibronectin
EDAvidin: Fusion protein between EDA and the N terminal end of streptavidin
DC: Dendritic cell
BMDC: Bone-marrow-derived DC
APC: Antigen presenting cell
NS3: Nonstructural protein 3 from hepatitis C virus.

## References

[1] Bonifaz L, Bonnyay D, Mahnke K, Rivera M, Nussenzweig MC, Steinman RM (2002). Efficient targeting of protein antigen to the dendritic cell receptor DEC-205 in the steady state leads to antigen presentation on major histocompatibility complex class I products and peripheral CD8+ T cell tolerance. *Journal of Experimental Medicine*.

[2] Cuadros C, Lopez-Hernandez FJ, Dominguez AL, McClelland M, Lustgarten J (2004). Flagellin fusion proteins as adjuvants or vaccines induce specific immune responses. *Infection and Immunity*.

[3] Hart JP, Gunn MD, Pizzo SV (2004). A CD91-positive subset of CD11c+ blood dendritic cells: characterization of the APC that functions to enhance adaptive immune responses against CD91-Targeted antigens. *Journal of Immunology*.

[4] Lasarte JJ, Casares N, Gorraiz M (2007). The extra domain A from fibronectin targets antigens to TLR4-expressing cells and induces cytotoxic T cell responses in vivo. *Journal of Immunology*.

[5] Tagliani E, Guermonprez P, Sepúlveda J (2008). Selection of an antibody library identifies a pathway to induce immunity by targeting CD36 on steady-state CD8*α*+ dendritic cells. *Journal of Immunology*.

[6] Tighe H, Takabayashi K, Schwartz D (2000). Conjugation of protein to immunostimulatory DNA results in a rapid, long-lasting and potent induction of cell-mediated and humoral immunity. *European Journal of Immunology*.

[7] Trumpfheller C, Finke JS, López CB (2006). Intensified and protective CD4+ T cell immunity in mice with anti-dendritic cell HIV gag fusion antibody vaccine. *Journal of Experimental Medicine*.

[8] Trumpfheller C, Longhi MP, Caskey M (2012). Dendritic cell-targeted protein vaccines: a novel approach to induce T-cell immunity. *Journal of Internal Medicine*.

[9] Wagner H (2009). The immunogenicity of CpG-antigen conjugates. *Advanced Drug Delivery Reviews*.

[10] Palucka K, Banchereau J (2012). Cancer immunotherapy via dendritic cells. *Nature Reviews Cancer*.

[11] Hawiger D, Inaba K, Dorsett Y (2001). Dendritic cells induce peripheral T cell unresponsiveness under steady state conditions in vivo. *Journal of Experimental Medicine*.

[12] Delneste Y, Magistrelli G, Gauchat JF (2002). Involvement of LOX-1 in dendritic cell-mediated antigen cross-presentation. *Immunity*.

[13] He LZ, Crocker A, Lee J (2007). Antigenic targeting of the human mannose receptor induces tumor immunity. *Journal of Immunology*.

[14] Brandão JG, Scheper RJ, Lougheed SM (2003). CD40-targeted adenoviral gene transfer to dendritic cells through the use of a novel bispecific single-chain Fv antibody enhances cytotoxic T cell activation. *Vaccine*.

[15] Kretz-Rommel A, Qin F, Dakappagari N (2007). In vivo targeting of antigens to human dendritic cells through DC-SIGN elicits stimulatory immune responses and inhibits tumor growth in grafted mouse models. *Journal of Immunotherapy*.

[16] Hemmi H, Akira S (2005). TLR signalling and the function of dendritic cells. *Chemical Immunology and Allergy*.

[17] Aranda F, Llopiz D, Díaz-Valdés N (2011). Adjuvant combination and antigen targeting as a strategy to induce polyfunctional and high-avidity T-cell responses against poorly immunogenic tumors. *Cancer Research*.

[18] Mansilla C, Berraondo P, Durantez M (2012). Eradication of large tumors expressing human papillomavirus E7 protein by therapeutic vaccination with E7 fused to the extra domain a from fibronectin. *International Journal of Cancer*.

[19] Mansilla C, Gorraiz M, Martinez M (2009). Immunization against hepatitis C virus with a fusion protein containing the extra domain A from fibronectin and the hepatitis C virus NS3 protein. *Journal of Hepatology*.

[20] Green NM (1990). Avidin and streptavidin. *Methods in Enzymology*.

[21] Howarth M, Chinnapen DJF, Gerrow K (2006). A monovalent streptavidin with a single femtomolar biotin binding site. *Nature Methods*.

[22] Pascolo S, Bervas N, Ure JM, Smith AG, Lemonnier FA, Pérarnau B (1997). HLA-A2.1-restricted education and cytolytic activity of CD8+ T lymphocytes from *β*2 microglobulin (*β*2m) HLA-A2.1 monochain transgenic H- 2Db *β*2m double knockout mice. *Journal of Experimental Medicine*.

[23] Mouriès J, Moron G, Schlecht G, Escriou N, Dadaglio G, Lederc C (2008). Plasmacytoid dendritic cells efficiently cross-prime naive T cells in vivo after TLR activation. *Blood*.

[24] Green NM, Toms EJ (1973). The properties of subunits of avidin coupled to sepharose. *Biochemical Journal*.

[25] Tacken PJ, De Vries IJM, Torensma R, Figdor CG (2007). Dendritic-cell immunotherapy: from ex vivo loading to in vivo targeting. *Nature Reviews Immunology*.

[26] van Montfoort N, Mangsbo SM, Camps MGM (2012). Circulating specific antibodies enhance systemic cross-priming by delivery of complexed antigen to dendritic cells in vivo. *European Journal of Immunology*.

[27] Khan S, Bijker MS, Weterings JJ (2007). Distinct uptake mechanisms but similar intracellular processing of two different toll-like receptor ligand-peptide conjugates in dendritic cells. *Journal of Biological Chemistry*.

[28] Sancho D, Mourão-Sá D, Joffre OP (2008). Tumor therapy in mice via antigen targeting to a novel, DC-restricted C-type lectin. *Journal of Clinical Investigation*.

[29] Steinman RM, Nussenzweig MC (2002). Avoiding horror autotoxicus: the importance of dendritic cells in peripheral T cell tolerance. *Proceedings of the National Academy of Sciences of the United States of America*.

[30] Ardeshna KM, Pizzey AR, Devereux S, Khwaja A (2000). The PI3 kinase, p38 SAP kinase, and NF-*κ*b signal transduction pathways are involved in the survival and maturation of lipopolysaccharide-stimulated human monocyte-derived dendritic cells. *Blood*.

[31] Rescigno M, Martino M, Sutherland CL, Gold MR, Ricciardi-Castagnoli P (1998). Dendritic cell survival and maturation are regulated by different signaling pathways. *Journal of Experimental Medicine*.

[32] Blander JM, Medzhitov R (2006). On regulation of phagosome maturation and antigen presentation. *Nature Immunology*.

[33] Sharma RK, Yolcu ES, Elpek KG, Shirwan H (2010). Tumor cells engineered to codisplay on their surface 4-1BBL and LIGHT costimulatory proteins as a novel vaccine approach for cancer immunotherapy. *Cancer Gene Therapy*.

