# Supplementary figures and images for: A Fusion Protein between Streptavidin and the Endogenous TLR4 Ligand EDA Targets Biotinylated Antigens to Dendritic Cells and Induces T Cell Responses In Vivo
Source: Biomed Res Int. 2013 Sep 5;2013:864720. doi: 10.1155/2013/864720 (PMC3777173; doi:10.1155/2013/864720)

**A**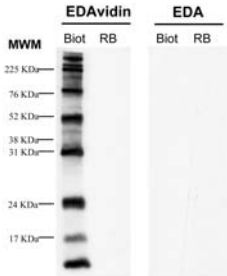**B**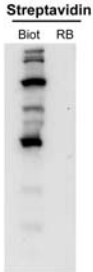

Supplement: Supplementary file 1 — Online Supplemental Material includes a western blot experiment to demonstrate the binding capacity of EDAvidin to biotinylated proteins (Supplementary Figure 1). Legend for Supplementary Figure 1: EDAvidin binds to biotinylated proteins. A molecular weight marker containing biotinylated proteins (M.W. 6,500-180,000, Sigma) (Biot), or the High-Range Rainbow Molecular Weight Marker (12000-225000, GE Healthcare) (RB) as negative control, were loaded into a 10% SDS-PAGE followed by electrophoretic transfer to nitrocellulose membranes. The detection of biotinylated proteins was carried out by incubating the membranes with 1,33 nmol of EDAvidin or EDA protein. After washing, the membranes were incubated with a rabbit polyclonal anti-EDA antibody. Membranes were them incubated with anti rabbit IgG horseradish-peroxidase (Cell Signalling), and developed by using ECL chemoluminescence system (Amersham). As a positive control, one of the membranes was incubated with a dilution 1/500 of horseradish peroxidase conjugated streptavidin. [file 864720.f1.pdf]
